# Combining yield potential and drought resilience in a spring wheat diversity panel

**DOI:** 10.1002/fes3.241

**Published:** 2020-09-18

**Authors:** Cara A. Griffiths, Matthew P. Reynolds, Matthew J. Paul

**Affiliations:** ^1^ Plant Science Rothamsted Research Harpenden UK; ^2^ International Maize and Wheat Improvement Centre (CIMMYT) Mexico City Mexico

**Keywords:** drought, genetic diversity, grain size and number, resilience, wheat, yield potential

## Abstract

Pressures of population growth and climate change require the development of resilient higher yielding crops, particularly to drought. A spring wheat diversity panel was developed to combine high‐yield potential with resilience. To assess performance under drought, which in many environments is intermittent and dependent on plant development, 150 lines were grown with drought imposed for 10 days either at jointing or at anthesis stages in Obregon, Mexico. Both drought treatments strongly reduced grain numbers compared with the fully irrigated check. Best performers under drought at jointing had more grain than poor performers, while best performers under drought at anthesis had larger grain than poor performers. Most high‐yielding lines were high yielding in one drought environment only. However, some of the best‐performing lines displayed yield potential and resilience across two environments (28 lines), particularly for yield under well‐watered and drought at jointing, where yield was most related to grain numbers. Strikingly, only three lines were high yielding across all three environments, and interestingly, these lines had high grain numbers. Among parameters measured in leaves and grain, leaf relative water content did not correlate with yield, and proline was negatively correlated with yield; there were small but significant relationships between leaf sugars and yield. This study provides a valuable resource for further crosses and for elucidating genes and mechanisms that may contribute to grain number and grain filling conservation to combine yield potential and drought resilience.

## Graphical abstract

Pressures of population growth and climate change require the development of resilient higher yielding crops, particularly to drought. To assess performance under drought, which in many environments is intermittent and dependent on plant development, 150 lines were grown with drought imposed for 10 days either at jointing or at anthesis stages in Obregon, Mexico. Several high‐yield lines across all drought trials were identified.

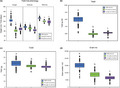

## 1 INTRODUCTION

Wheat (*Triticum aestivum* L.) is one of the three major food crops providing about a fifth of global dietary energy and protein (Ray, Mueller, West, & Foley, 2013). With continuing population growth and climate change and climate unpredictability, there is a pressing need to develop wheat varieties that combine higher yield potential with resilience, particularly to drought, the most widespread and significant abiotic stress (Godfray et al., 2010; Tilman, Balzer, Hill, & Befort, 2011). Drought is a complex stress, which can be acute or chronic, with drought resistance in plants falling into different categories, such as drought‐avoidant or drought‐tolerant (Yue et al., 2006). Biotechnology has offered promise in producing drought resilience in the laboratory, but biotech crops have yet to enter agriculture in a big way (Nuccio et al., 2015), and it is possible that GM or gene‐edited wheat may never be accepted. For traits such as yield potential and drought resilience, single‐gene solutions are likely to be very rare. Alternative non‐GM methodologies such as chemical approaches involving topical sprays such as trehalose‐6‐phosphate may at some stage be successful in the field (Griffiths et al., 2016). However, a mainstay of wheat improvement is the development of germplasm that combines high‐yield potential with resilience (Trethowan, Reynolds, Ortiz‐Monasterio, & Ortiz, 2007).

Genetic solutions to drought resilience are likely to depend on the drought environment. For example, a good level of earliness is an effective strategy for enhancing yield stability where wheat is exposed to terminal drought (Cattivelli et al., 2008), whereas late heading and flowering followed by a short grain filling period can be associated with higher yield when drought is experienced early in the season (van Ginkel et al., 1998; Shavrukov et al., 2017). A more xerophytic breeding strategy to limit evapotranspiration can be applied in more extreme environments, assuming economic viability. Many agricultural environments such as in North America and Europe experience mild‐to‐moderate drought conditions, which may occur at any time during the season. Traits that enable tolerance to drought at different developmental stages particularly during reproductive development (Senapati, Stratonovitch, Paul, & Semenov, 2018) combined with high‐yield potential would be a valuable inclusion into germplasm.

As a breeding strategy, selecting for high‐yield potential has frequently led to yield improvements under drought (Araus, Slafer, Reynolds, & Royo, 2002), particularly mild‐to‐moderate drought (Slafer et al., 2015; Tambussi, Nogués, Ferrio, Voltas, & Araus, 2005). This has been true for wheat and rice (Trethowan, van Ginkel, & Rajaram, 2002) and barley (Rizza et al., 2004). There are also some examples where direct selection for stress resistance was effective in improving yield (Bänziger, Edmeades, & Lafitte, 1999; Morgan, 2000). However, further progress in improving yield under drought will require the reduction of the gap between yield potential and yield in drought‐prone environments. This will depend on the introduction into high‐yielding genotypes traits that can improve drought tolerance without detrimental effects on yield potential.

Crop yields can be limited by many factors, such as availability of water and light, as well as key metabolic processes such as carbon metabolism. The plant's ability to reallocate and utilize carbon resources for growth and storage during development has a potentially strong influence on “source” and “sink” balance. Phloem loading, unloading, and transport of metabolites such as sugars have been shown to have a dynamic relationship with physiological status (Turgeon, 1996), and transport of carbohydrates has been implicated as key targets for improving crop productivity (Bihmidine, Hunter, Johns, Koch, & Braun, 2013; Griffiths et al., 2016). Total sugars have been previously shown to accumulate during wheat drought stress (Abid et al., 2018) and carbohydrate remobilization during drought stress, and recovery from drought is thought to preserve crop yields (Blum, 2005; White, Rogers, Rees, & Osborne, 2015). Carbohydrate accumulation is associated with osmotic adjustment alongside other compatible solutes such as free amino acids and proline during drought stress (Molinari et al., 2007). Proline has been used as a marker for plant stress in many studies due to its accumulation in many plant tissues during abiotic stress exposure (reviewed in Per et al., 2017). Proline is known to stabilize the antioxidant system through osmotic adjustments to diminish effects of reactive oxygen species (ROS) and to protect cell membrane integrity during abiotic stress (Reddy et al., 2015).

To identify lines that may be suitable for development of high yield and resilience and any associated metabolic characteristics related to drought‐ and yield‐related traits in this study, we subjected a bread wheat diversity panel developed and selected for drought and heat tolerance. The panel was subjected to acute drought stress at the jointing and anthesis growth stages, toward the beginning and end of the period of reproductive development, the period particularly sensitive to drought. Differences in panel individuals were assessed for yield and yield parameters such as grain numbers and thousand grain weight and plant growth traits and other drought‐related components proline, sugars, and water content. These yield components and metabolite accumulations were measured to assess the broad impact of drought stress on these wheat varieties on these factors and uncover potential markers for drought resilience. The results presented provide insight into yield diversity associated with drought within the bread wheat panel, which are discussed within context of selecting new material that perform well under two environments (15 lines out of 150) and rarely in three environments (three lines out of 150) in addition to insight into the possibility of providing metabolic markers and breeding backgrounds for improved drought tolerance for future wheat breeding strategies.

## 2 MATERIALS AND METHODS

### 2.1 Bread wheat diversity panel

The panel was assembled from spring wheat (*T. aestivum*) sources after screening approximately 60,000 lines for heat and drought adaption in the Sonoran Desert. They were derived from the following sources: (a) International nurseries where every year approximately 1,000 new, high‐yielding, disease‐resistant wheat lines with appropriate end‐use quality are generated by CIMMYT and delivered via IWIN to most public and private wheat improvement programs worldwide, where they are tested at approximately 200 sites annually. (b) Wheat with ancestral chromosomal introductions. Many elite lines already contain alien introgressions from the Triticeae tribe that are linked to improved yield potential and disease resistance (Ortiz et al., 2008). CIMMYT's GenBank holds around 500 accessions with specific translocations (e.g., 7Ag.7Dl, 1B.1R, Lr34, Lr42), while thousands of elite lines are derived from crosses with synthetic wheat. (c) Landraces. Approximately 15,000 spring wheat landraces from the World Wheat Collection have recently been prescreened under high temperature stress (Reynolds et al., 2015). Several hundred landraces were selected for superior yield and biomass. (d) Focused Identification of Germplasm Strategy (FIGS) is a landrace panel that has been selected based on their origin being in regions with abiotic stress (Sehgal et al., 2015). A selection of 150 lines from this panel was used in this study, based on the appearance of chlorophyll retention/turgidity during drought at the jointing stage, from a subset of 300 individuals consistently displaying drought‐tolerant characteristics. Each plot was assessed on a scale of 1–5 based on appearance of nonstressed wheat, from this, 50 very stressed, 50 moderately stressed, and 50 nonstressed individuals were chosen.

### 2.2 Field experiments

To assess the diversity panel for promising germplasm for yield under drought combined with high‐yield potential, 150 lines were grown in successive seasons with drought imposed for approximately 10 days at jointing (abbreviated as DJ) (Zadoks GS30), or the beginning of anthesis (abbreviated as DA) (Zadoks GS60) in consecutive seasons (2016, 2017) at the CIMMYT field site at Obregon, Mexico. Yields and yield parameters were compared with yield under full irrigation (2016), both trials were sown in mid‐November, 9 November 2015 (for the 2016 trials), and 12 November 2016 (for the 2017 trial). For the jointing trial, drought treatment ended at GS33, and for the anthesis trial, GS65. The two developmental stages were chosen because of the likely contrasting effects of drought on the two basic processes of yield determination, grain number, and grain size due to drought at these times (Dolferus, Ji, & Richards, 2011). To combat heterogeneity in the field, all drought stress treatments were commenced within 3 days of the average time each panel individual took to reach jointing or anthesis.

**FIGURE 1:**
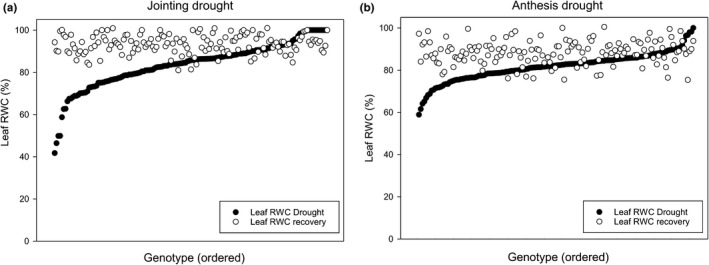
Leaf relative water contents both at drought and at drought recovery. Leaf relative water contents (RWC) in the drought at jointing (a) and drought at anthesis (b) field trials. Closed circles are leaf RWC after 10 days of drought, and open circles are leaf RWC 1 day after rewatering. Individuals are placed in ascending order based on leaf RWC at drought, *n* = 150

Field trials were conducted in Mexico CIMMYT Obregon Experimental Station, Yaqui valley, Sonora, north‐western Mexico, 27°25′N, 109°54′W, 38 m above sea level in 2016 and 2017 with drip irrigation. All experiments were sown in an α‐lattice design with two replicates with a plot size of 0.5 × 1.6 m with a 10 g/m^2^ seed density. All plots were fertilized at sowing (200:40:0, N:P:K). Trials in 2016 had an average temperature of 20.22°C, 0.14 mm rainfall, 63.47% relative humidity, and 499.36 W/m^2^ radiation. Trials in 2017 had an average temperature of 19.12°C, 0.28 mm rainfall, 68.08% relative humidity, and 473.76 W/m^2^ radiation. Drought was imposed by withholding irrigation to achieve significant dehydration (Figure 1) either at the jointing stage (2016) or at anthesis stage (2017). Well‐watered trial received irrigation throughout.

### 2.3 Phenotypic measurements

Phenotypic traits measured in this study were grain yield (GY), thousand grain weight (TGW), grain number (GNO), days to heading (DH), plant height (PH), days to anthesis (DA), and days to maturity (DM). Grain yield was measured by weighing the grain harvested from the middle of each plot in grams. Average kernel weight was estimated by weighing 200 randomly selected grains per plot in grams. Grain number was calculated by dividing yield by average kernel weight. DH was recorded as the number of days from trial emergence until 50% of the spikes in each plot had emerged completely from the flag leaf. PH was measured by the average of three values per plot in centimeters from the soil surface to the tip of the spike excusing awns. DM was measured as days from trial emergence until 50% of the spikes per plot had reached senescence.

### 2.4 Sampling

For both trials, samples were taken between 10:00 and 12:00 hr 10 days after drought imposition, and again 1 day after rewatering. Three fully unfurled source leaves were taken from each plot during the jointing drought trial (2016), and three flag leaves and three ears per plot were taken during the anthesis drought trial (2017) at both drought and rewatered time points. Samples were immediately snap‐frozen in liquid N_2._ Leaf relative water content (RWC) was measured as fresh weight and calculated as described below. All sugar, proline, RWC, and chlorophyll data per genotype were analyzed as averages of 2 × 3 pooled samples per plot.

### 2.5 Relative water content measurement

RWC was measured using three steps. Leaf fresh weight (FW) was taken by weighing the midsection of harvested leaf. Turgid weight (TW) of the same leaf midsection was determined after floating the leaf in a petri dish of distilled water for 16 hr. Leaf tissue dry weight (DW) was determined after leaf drying for 16 hat 60°C. The following equation was used to determine RWC as a percentage: [(FW − DW)/(TW − DW)] × 100.

### 2.6 Sugar, proline, and chlorophyll measurements

All samples from both trials were ground to a fine powder in liquid N_2_, and 100–150 mg of tissue was extracted in 1–1.5 ml (relative to sample weight) 80% ethanol in screw‐capped tubes for 2 hr with shaking at 80°C. Samples were cooled to room temperature (RT) and centrifuged at 12,000 *g* at RT for 5 min to remove debris. A 10 µl aliquot of sugar extract was subject to coupled enzyme assay to determine glucose, fructose, and sucrose concentration. The enzyme assay was conducted at RT in a HEPES‐based buffer (100 mM HEPES, 4 mM MgCl_2_, 1 mM NAD+, 0.5 mM ATP) at pH 7.4 using 10 µl of ethanolic sugar extract. Enzymes were added sequentially for determination of each hexose sugar as follows: (a) Glucose was measured 30 min after enzyme addition of 1.5U hexokinase and 1.2U NAD‐dependent glucose‐6‐phosphate dehydrogenase. (b) Fructose was measured by addition of 0.2U of phosphoglucoisomerase postglucose reaction for 30 min. (c) Sucrose was measured 1.5 hr after addition of 10U invertase. Measurements of each sugar were completed on a microplate reader by measuring the reduction of NAD+ to NADH at 340 nm (Molecular Devices Spectramax). Concentration of each sugar per sample was determined using a standard curve and standardized to weight of tissue extracted in mg. Proline was measured using the acetic acid‐ninhydrin method described in Giannakoula, Moustakas, Syros, and Yupsanis (2010) using ethanolic extracts from the same sample extract described above. Leaf chlorophyll content was measured in leaf midsections excluding the midrib by extracting in cold methanol described by Ritchie (2006).

### 2.7 Statistical analysis

All statistical analysis was completed using SigmaPlot version 14.0 and Genstat *for Windows* 19th edition software. Yield and yield components were analyzed using a general linear model (GLM) approach. Means were adjusted for phenological effects (anthesis time, plant height, maturity) and relative water content (RWC) in all trials using GLM. All biochemical data (glucose, fructose, sucrose, proline, and chlorophyll) were adjusted for RWC as covariate. General differences between phenology and yield of each trials were measured using ANOVA. High‐yielding lines were identified using ANOVA on ranks using the Bonferroni correction (0.05/*N*, where *N* is the number of genotypes tested). PCA was conducted based on the correlation matrix, with the chi‐square test to assess significance of the PCA. PCA was performed on high‐yielding lines (*n* = 50) and low‐yielding lines (*n* = 45 separately). To describe the magnitude of relationships between specific sugars glucose, fructose, and sucrose and yield components, Pearson's product–moment correlation coefficients were calculated separately and together (jointing drought samples, jointing recovery samples, anthesis drought samples, anthesis recovery samples).

## 3 RESULTS

### 3.1 Drought affects leaf RWC plant phenology and reduces yield

**FIGURE 2:**
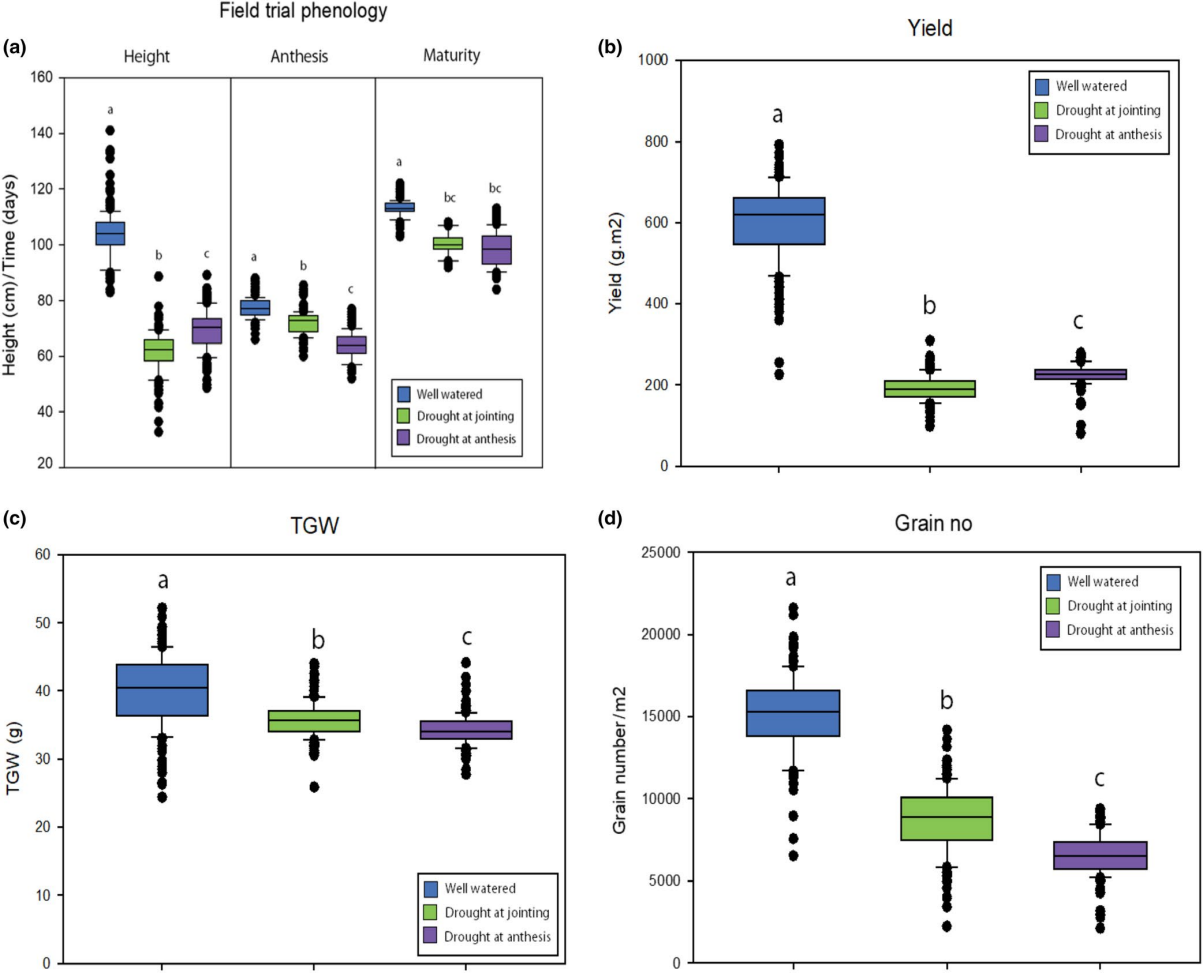
Crop phenology and yield across all trials. Average height, anthesis time, and maturity time across all trials (a), average final yield (b), average TGW (c), and average grain number (d) for all trials. Whiskers indicate data variation within the group (*n* = 150), lettering denotes significance (*p* < .001)

Drought at jointing and anthesis caused varying effects on leaf RWC across the panel at drought, and all individuals were able to restore leaf RWC once drought was relieved (Figure 1). Differences are seen in the capacity of the source leaves to retain water during drought in the jointing (Figure 1a) and anthesis (Figure 1b) trial. There was greater variation in the RWC in leaves where drought was imposed at jointing compared with drought imposed at anthesis, with some genotypes having below 40% RWC after drought at jointing compared with a minimum level just below 60% after drought at anthesis. Drought at jointing and anthesis both significantly reduced plant height, time to anthesis, and maturity time. However, time to maturity was not significantly different between both drought treatments (Figure 2a). Drought at both jointing and anthesis significantly reduced yield, from an average of 604 g/m^2^ in well‐watered conditions to 192 and 225 g/m^2^ in drought at jointing and drought at anthesis trials, respectively (Figure 2b). Similar trends are seen in TGW and grain number measurements where averages of TGW and grain number between well‐watered and drought at jointing or anthesis were significantly reduced. In the case of TGW, averages of 40 g in well‐watered conditions were reduced to 35 g drought at jointing and 34 g in drought at anthesis trials (Figure 2c). Grain numbers were reduced from an average of 15,226 seeds/m^2^ in well‐watered trials to 8,604 and 6,585 seeds/m^2^ in drought at jointing and drought at anthesis trials, respectively (Figure 2d).

**FIGURE 3:**
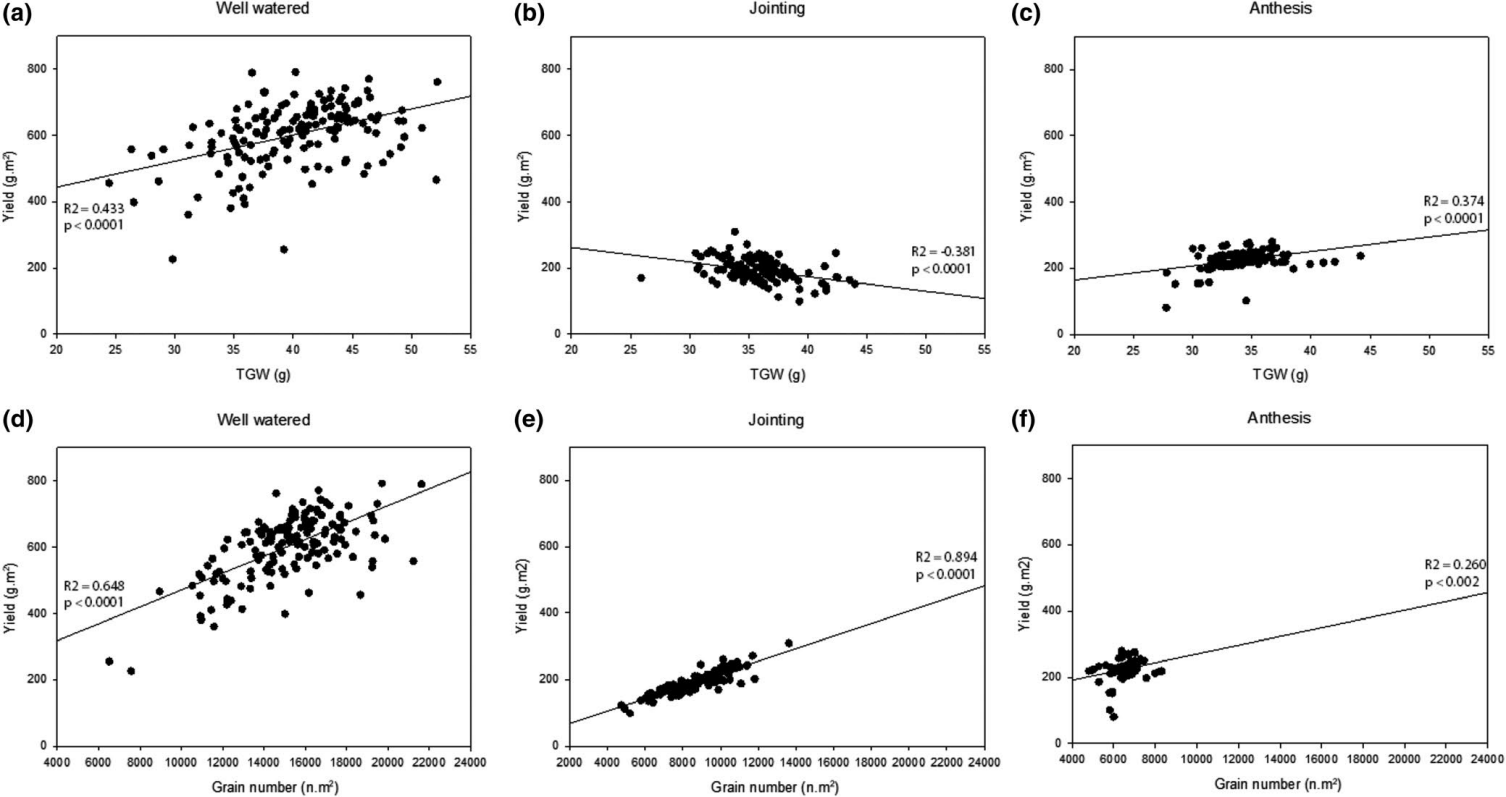
Relationship between final yield, TGW, and grain number. Pearson's product–moment correlations between yield and TGW (a‐c) and yield and grain number (d‐f) in well‐watered, drought at jointing and drought at anthesis trials. Line denotes trendline, *n* = 150

The relationships between final yield, TGW, and grain number show differences in each trial. In well‐watered trials, the relationship between yield and TGW, and that between yield and grain number are both positive showing a contribution of both grain weight and grain number to final yield (Figure 3a,d). After drought treatment at jointing these relationships change, where a small but significant relationship shows that during drought at jointing TGW has a reduced contribution to final yield (Figure 3b), while a strong positive relationship between final yield and grain number shows grain numbers have a greater contribution to final yields (Figure 3e). After drought during anthesis, small but significant relationships exist between final yields and TGW (Figure 2c) and final yield and grain number (Figure 2f), suggesting that similar to well‐watered trials, both TGW and grain number contribute to higher final yields after drought during anthesis.

Analysis of phenology effects on final yield components (Table S1) in each trial reveals that in the well‐watered trial yield, grain number and TGW are influenced by time to anthesis and maturity time, while plant height only influences final yield. When drought is applied at jointing, yield and grain number correlate together and are negatively correlated with anthesis time and TGW, while plant height and maturity time are negatively correlated, plant height positively correlates with TGW. When drought is applied at anthesis, TGW and yield are correlated together, and TGW and yield are negatively correlated with maturity time. Height and time to anthesis have some effect on grain number.

### 3.2 High‐yielding lines occur simultaneously in well‐watered and drought environments

**FIGURE 4:**
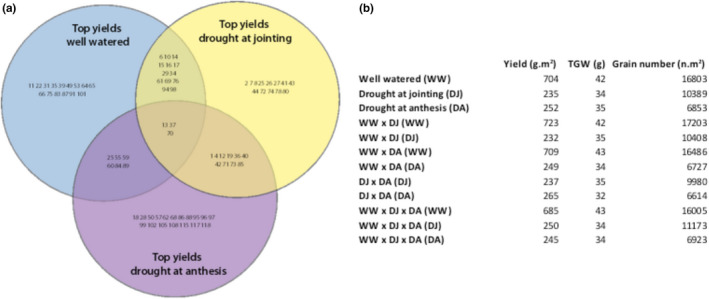
Top‐performing lines across all trials. Highest yielding lines with drought at jointing, drought at anthesis, and well‐watered field trials and overlap between trials (a), table of yield, TGW, and grain number averages of each group (b). Venn intercepts are represented by: trial type x trial type in table, adjacent brackets indicate the trial data shown

Individual lines were ranked according to yield after drought at jointing or anthesis compared with well‐watered conditions (Tables S2–S4), and based on average yield across all conditions, these top‐yielding lines are significantly higher yielding than low‐ranked performers (*p* < .001). Overlap of high‐yielding lines in well‐watered (WW) and drought at jointing (DJ), well‐watered and drought at anthesis (DA), and DA and DJ are also observed (Figure 4a,b). Three individuals were in the top highest yielding lines under all conditions (13, 37, 70) (Figure 4a,b). These three varieties with average yields in well‐watered, drought and jointing and drought at anthesis 685, 250, and 242 g/m^2^, respectively were observed. Interestingly, high‐yielding individuals in all three conditions had higher grain numbers in comparison with high‐yielding individuals across one or two conditions (Figure 4b).

### 3.3 Sugars and proline are correlated with final yield, grain number, and TGW

To observe the relationships between sugars, proline, and chlorophyll content on yield and yield components, a PCA was conducted. In high‐yielding lines after drought at jointing, PCA (Table 1) grouped the data into four main components, which together accounted for 71.62% of the data. PC1 shows positive correlations between total leaf sugars at recovery and TGW, which are negatively correlated with yield and grain numbers, while PC2 shows medium to strong correlations between total leaf sugars and chlorophyll at drought. PC3, PC4 and PC5 show correlations between proline and sugars at recovery and proline and total chlorophyll but show no strong correlations of these parameters with yield, TGW, or grain numbers. In low‐yielding lines after drought at jointing, PCA grouped the data into 5 main components, representing 80.16% of the data (Table 2). In contrast to high‐yielding lines, PC1 shows correlations between total leaf sugars and chlorophyll in both drought and recovery, and positive associations with grain numbers, but negative associations with TGW. PC2 shows correlations between total sugars and chlorophyll at drought with negative correlations with grain number and yield, while PC3 showed weaker correlations between TGW and leaf proline content at recovery and negative correlations with leaf chlorophyll at recovery. PC4 showed that leaf proline contents at drought and recovery are negatively correlated with TGW.

**TABLE 1:** Eigenvalues of the correlation matrix for PCA of yield, yield components, sugar, proline, and chlorophyll data in drought at jointing in high‐yielding lines

	PC 1	PC 2	PC 3	PC 4	PC 5	PC 6	PC 7	PC 8
Yield (g/m^2^)	−0.467	0.223	−0.259	−0.133	0.205	0.468	−0.00502	0.367
Grain number (n/m^2^)	−0.553	0.225	0.0151	−0.0657	0.128	0.0376	0.191	0.0694
TGW (g)	0.469	0.176	0.22	0.0544	0.131	0.435	−0.432	0.457
Soluble sugar drought (mg/g dwt)	0.138	0.593	0.0229	0.0738	−0.197	−0.525	0.251	0.482
Soluble sugar recovery (mg/g dwt)	0.363	−0.233	−0.306	0.129	0.508	0.0845	0.635	0.167
Proline drought (mmol/g dwt)	0.143	0.352	−0.552	−0.185	0.424	−0.265	−0.415	−0.302
Proline recovery (mmol/g dwt)	0.198	−0.045	0.0201	−0.942	−0.173	0.0563	0.182	0.0498
Chlorophyll drought (mg/g dwt)	0.207	0.56	0.0333	0.116	−0.203	0.463	0.321	−0.52
Chlorophyll recovery (mg/g dwt)	0.0582	−0.154	−0.696	0.136	−0.613	0.143	−0.0526	0.167
Eigenvalue	2.473	1.751	1.259	0.962	0.769	0.619	0.571	0.397
Proportion (%)	27.481	19.461	13.988	10.692	8.541	6.877	6.346	4.412
Cumulative (%)	27.481	46.942	60.93	71.622	80.163	87.04	93.387	97.799

Note

Only significant principal components are shown (*p* < .01), *n* = 50.

**TABLE 2:** Eigenvalues of the correlation matrix for PCA of yield, yield components, sugar, proline, and chlorophyll data in drought at jointing in low‐yielding lines

	PC 1	PC 2	PC 3	PC 4	PC 5	PC 6	PC 7	PC 8
Yield (g/m^2^)	0.276	−0.535	0.137	0.052	0.257	−0.156	−0.309	0.218
Grain number (n/m^2^)	0.353	−0.483	0.133	−0.107	0.222	−0.292	0.0631	−0.228
TGW (g)	−0.301	0.178	0.213	0.553	0.578	0.0811	−0.373	−0.0652
Soluble sugar drought (mg/g dwt)	0.423	0.438	0.194	0.0977	0.106	−0.279	0.0429	0.00378
Soluble sugar recovery (mg/g dwt)	0.442	−0.0774	0.00225	0.183	0.184	0.79	0.328	0.00177
Proline drought (mmol/g dwt)	0.00453	0.0737	0.692	−0.433	−0.205	0.324	−0.415	−0.0302
Proline recovery (mmol/g dwt)	−0.0299	0.237	−0.255	−0.665	0.653	0.0422	0.00352	0.0236
Chlorophyll drought (mg/g dwt)	0.368	0.106	−0.546	0.0302	−0.191	0.13	−0.686	−0.0581
Chlorophyll recovery (mg/g dwt)	0.445	0.422	0.194	0.0854	−0.0235	−0.244	0.0947	0.0462
Eigenvalue	2.499	2.191	1.274	1.073	0.74	0.623	0.457	0.111
Proportion (%)	27.771	24.348	14.153	11.924	8.219	6.927	5.081	1.233
Cumulative (%)	27.771	52.119	66.273	78.197	86.416	93.342	98.423	99.656

Note

Only significant principal components are shown. (*p* < .01), *n* = 45.

The relationships of sugars, proline, and chlorophyll with yield, TGW, and grain number during drought at anthesis in high‐yielding lines include both leaf data and grain data for PCA (Table 3), which grouped the data into six main principal components, which together accounted for 69% of the data. PC1 showed correlations between grain proline and sugars during recovery, while PC2 showed correlations between leaf and grain sugars during drought with TGW, but were negatively correlated with grain proline and leaf chlorophyll during drought alongside yield. PC3 showed positive correlations between leaf proline at drought and recovery and TGW, with negative correlations between grain proline at recovery and grain number. Further principal components contained leaf proline and chlorophyll at recovery, yield, grain number, and TGW (PC4); leaf proline during drought and recovery, grain proline during recovery, leaf chlorophyll at recovery, and grain sugars during drought (PC5); and leaf proline and chlorophyll at recovery, grain number, and TGW (PC6). In low‐yielding lines, PCA grouped the data into five main principal components representing 63.35% of the data (Table 4). PC1 show correlations between yield, grain number, and TGW, which negatively correlated with total grain sugars at drought, while PC2, PC3, PC4, and PC5 show relationships between leaf and grain sugars, proline, and chlorophyll, but no relationships with yield, TGW, or grain numbers.

**TABLE 3:** Eigenvalues of the correlation matrix for PCA of yield, yield components, sugar, proline, and chlorophyll data in drought at anthesis in high‐yielding lines

	PC 1	PC 2	PC 3	PC 4	PC 5	PC 6	PC 7	PC 8
Yield (g/m^2^)	0.116	−0.4240	0.276	0.326	−0.0223	−0.114	0.434	−0.245
Grain number (n/m^2^)	−0.147	0.0547	−0.45	0.302	−0.233	0.454	0.298	0.234
TGW (g)	0.0179	0.1690	0.322	0.404	0.199	0.441	−0.149	0.433
Leaf soluble sugar drought (mg/g dwt)	0.209	0.3700	0.0896	0.289	0.0607	0.283	0.0871	−0.643
Leaf soluble sugar recovery (mg/g dwt)	0.51	0.0794	0.149	−0.133	−0.238	−0.0216	−0.103	0.26
Grain soluble sugar drought (mg/g dwt)	0.256	0.4680	0.0743	−0.00348	−0.312	−0.143	0.209	−0.142
Grain soluble sugar recovery (mg/g dwt)	0.523	−0.0074	−0.238	0.146	0.0883	0.0983	−0.144	0.0956
Leaf proline drought (mmol/g dwt)	0.00945	−0.0449	0.501	0.195	0.393	−0.11	0.027	0.123
Leaf proline recovery (mmol/g dwt)	0.296	−0.2490	0.315	−0.315	−0.365	0.305	−0.099	0.0272
Grain proline drought (mmol/g dwt)	0.287	−0.4110	−0.176	−0.142	0.22	0.204	0.474	0.0358
Grain proline recovery (mmol/g dwt)	0.362	0.1530	−0.318	−0.0635	0.535	−0.229	−0.0254	0.0584
Leaf chlorophyll drought (mg/g dwt)	0.0685	−0.3940	−0.179	0.235	−0.0178	0.137	−0.617	−0.351
Leaf chlorophyll recovery (mg/g dwt)	−0.128	0.1210	0.104	−0.548	0.344	0.508	0.0243	−0.212
Eigenvalue	2.137	1.711	1.588	1.347	1.233	0.996	0.937	0.869
Proportion (%)	16.436	13.16	12.213	10.362	9.484	7.66	7.204	6.687
Cumulative (%)	16.436	29.596	41.809	52.171	61.655	69.315	76.519	83.206

Note

Only significant principal components are shown. (*p* < .01), *n* = 50.

**TABLE 4:** Eigenvalues of the correlation matrix for PCA of yield, yield components, sugar, proline, and chlorophyll data in drought at anthesis in low‐yielding lines

	PC 1	PC 2	PC 3	PC 4	PC 5	PC 6	PC 7	PC 8
Yield (g/m^2^)	0.392	0.0459	−0.197	0.205	0.00725	0.499	0.00483	−0.169
Grain number (n/m^2^)	0.539	0.0296	−0.0253	−0.172	0.0551	0.102	−0.132	0.375
TGW (g)	0.561	0.0913	0.0272	−0.039	−0.0143	0.147	0.207	0.111
Leaf soluble sugar drought (mg/g dwt)	−0.261	0.136	0.29	−0.103	−0.127	0.632	0.212	0.00717
Leaf soluble sugar recovery (mg/g dwt)	−0.101	0.322	0.34	0.476	0.0883	0.259	−0.173	0.0499
Grain soluble sugar drought (mg/g dwt)	−0.305	0.0434	−0.318	0.237	0.354	0.173	0.389	0.352
Grain soluble sugar recovery (mg/g dwt)	0.0381	0.427	−0.183	0.386	0.128	−0.212	−0.42	0.2
Leaf proline drought (mmol/g dwt)	0.0271	−0.557	0.0644	0.255	−0.212	0.0186	0.13	0.338
Leaf proline recovery (mmol/g dwt)	0.204	0.232	0.173	0.314	−0.0191	−0.399	0.69	−0.0913
Grain proline drought (mmol/g dwt)	−0.0094	−0.277	−0.353	0.0114	0.567	0.0877	0.0395	0.0314
Grain proline recovery (mmol/g dwt)	−0.0167	0.0354	0.543	−0.278	0.366	−0.103	−0.0266	0.5
Leaf chlorophyll drought (mg/g dwt)	0.0614	−0.456	0.24	0.491	−0.112	−0.0111	−0.198	0.0568
Leaf chlorophyll recovery (mg/g dwt)	−0.146	0.182	−0.339	−0.052	−0.564	0.0013	0.0457	0.524
Eigenvalue	2.298	1.926	1.46	1.336	1.215	1.143	0.8	0.748
Proportion (%)	17.676	14.819	11.231	10.277	9.347	8.794	6.157	5.754
Cumulative (%)	17.676	32.495	43.727	54.004	63.351	72.144	78.302	84.055

Note

Only significant principal components are shown (*p* < .01), *n* = 45.

**FIGURE 5:**
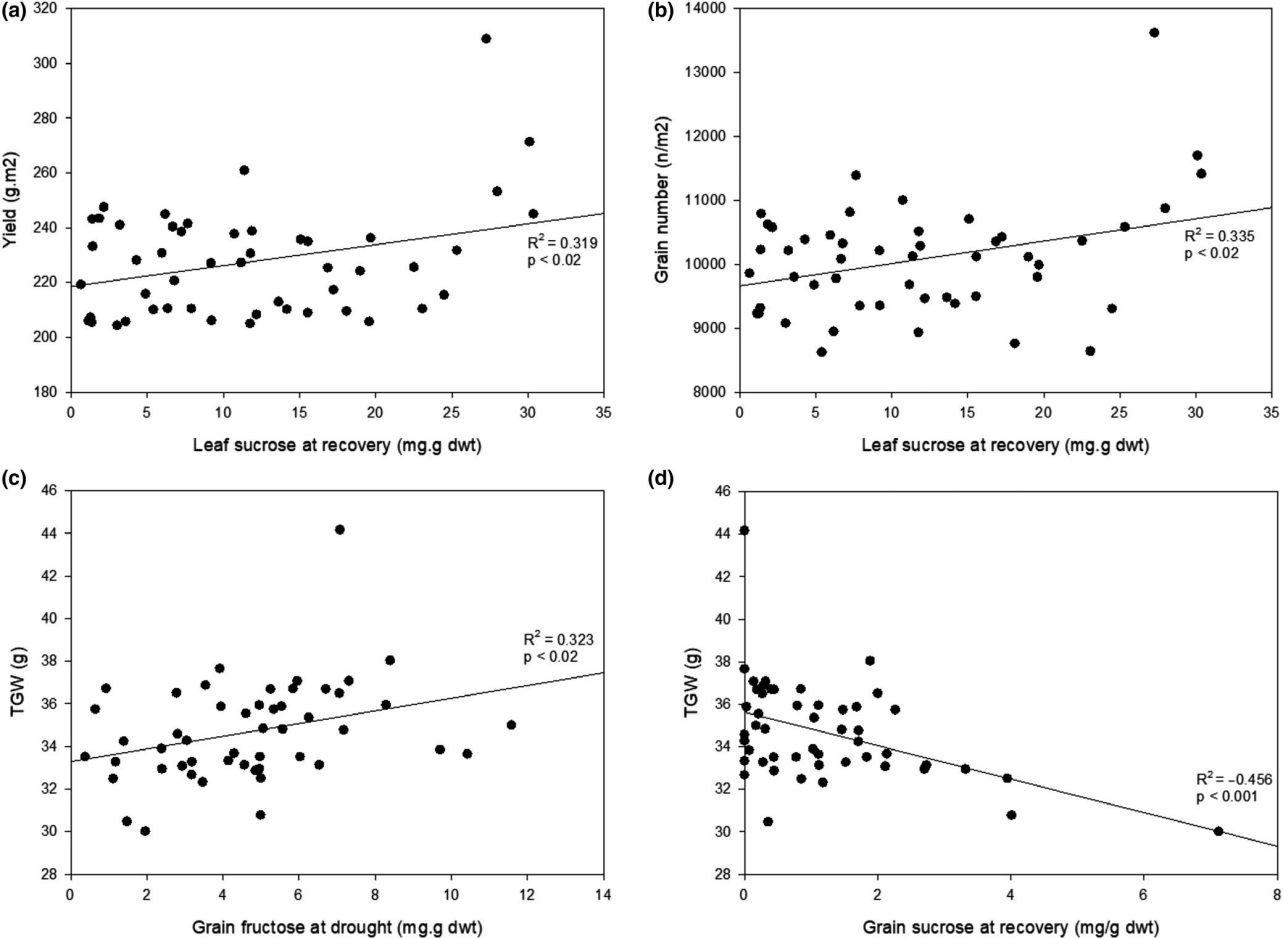
Relationship between yield components and sugars during drought and recovery in high‐yielding lines. Pearson's product–moment correlations between yield and leaf sucrose at recovery (a) and grain number and leaf sucrose at recovery (b) after drought at jointing, and TGW and grain fructose at drought (c) and sucrose a recovery (d) after drought during anthesis. Line denotes trendline, *n* = 50

**FIGURE 6:**
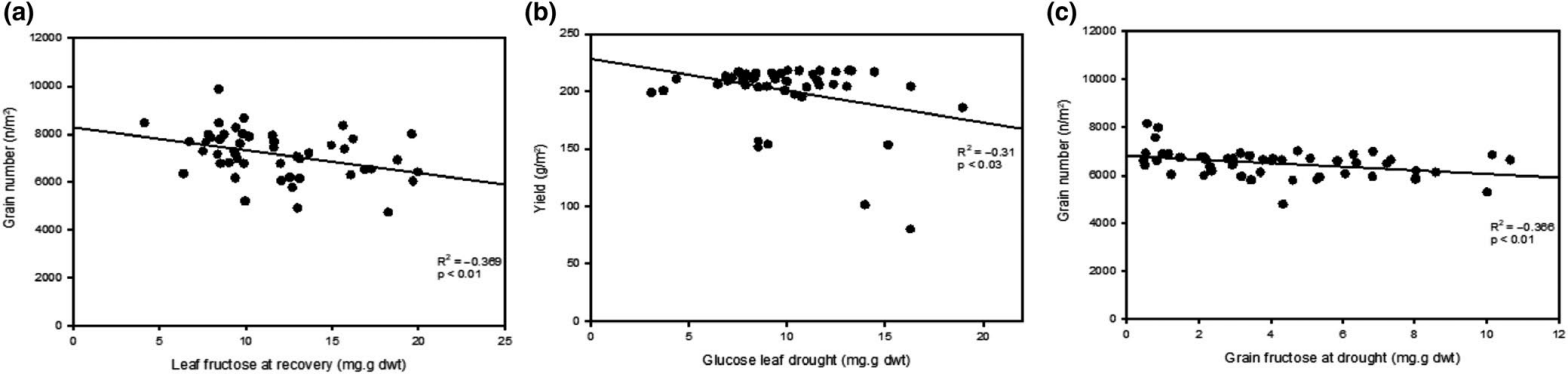
Relationship between yield components and sugars during drought and recovery in low‐yielding lines. Pearson's product–moment correlations between grain number and leaf fructose at recovery after drought at jointing (a), yield and leaf glucose at drought (b), and grain number and grain fructose at drought (c) after drought at anthesis. Line denotes trendline, *n* = 45

Based on the outcome of the PCA, Pearson's correlations between yield, TGW, and grain number and specific soluble sugars glucose, fructose, and sucrose were analyzed in high‐yielding lines (Figure 5). During drought at jointing, there was a mild yet significant correlation between leaf sucrose content at recovery and final yields and grain numbers (Figure 5a,b) both of which are positively correlated (*p* < .02). During drought at anthesis, grain fructose content during drought and grain sucrose content during recovery had mild but significant correlations with TGW (*p* < .02, *p* < .001, respectively) (Figure 5c,d). Correlations between specific soluble sugars, yield, and grain number were different in low‐yielding lines, where during drought at jointing, leaf fructose at recovery is negatively correlated with grain number (Figure 6a). During drought at anthesis, both yield and leaf glucose (Figure 6b) and grain number and grain fructose (Figure 6c) were negatively correlated.

## 4 DISCUSSION

Combining yield potential with resilience is an important goal for productive wheat yields in most parts of the world. A challenge in the development of drought resilience in many environments is the unpredictability of water availability. Hence, resilience to drought at more than one developmental stage is highly desirable (Khadka, Earl, Raizada, & Navabi, 2020). In this study, a bread wheat panel with combined genetic diversity for yield potential and drought tolerance trialed at Obregon, Mexico, was found to exhibit a large range of genetic variation for high yields after drought at jointing, drought at anthesis, and yield potential both individually and in combination. This population will be valuable in enabling selection for combining drought resilience at more than one growth stage with yield potential and for the determination of genes and mechanisms that facilitate high yields and yield stability.

Among the best‐performing lines under drought at jointing, drought at anthesis, or under full irrigation, 72% were high yielding in that environment only (Figure 4a) with some of these lines performing relatively poorly under the other drought condition or under full irrigation. This emphasizes the complexity of drought resilience, and that its effects on yield are highly dependent on developmental stage (Senapati et al., 2018; Yu, Zhang, Sun, & Song, 2018). It also shows that selecting for yield potential, a strategy that has also resulted in combined selection for drought resilience (Slafer et al., 2015; Tambussi et al., 2005), while having some utility, is not a straightforward way for producing resilience to drought at both jointing and anthesis stages.

The study highlights the differences in how plant yield and yield component priorities change in response to drought at different growth stages, where drought at jointing will favor grain number preservation to maintain yield, while drought at anthesis will favor TGW preservation to maintain yield (Figures 2, 3), an important contributing factor to the separation of resilience to drought at jointing from drought at anthesis (Blum, 1997). Both drought treatments reduced grain numbers, but there was an important distinction between the two situations. The best‐performing lines after drought at jointing were lines with the most grains, and hence were able to resist to a greater extent the pressure of drought to reduce grain set, while worst‐performing lines under this condition with the fewest grains increased grain size to a small extent (<10%). This did not, however, compensate for the loss of yield due to grain numbers. Under drought at anthesis, grain numbers were also reduced, but in this situation, best‐performing lines did not have more grain than worst‐performing lines but instead were able to increase grain size of the remaining grain more than the worst‐performing lines by about 25%; hence, TGW was greater. Yield potential was due mainly to grain numbers (100% difference between high and low performers) but also to grain size (47% difference between high and low performers). Grain numbers have been a major determinant of wheat yield improvement (Dolferus et al., 2011), and grain numbers play the most significant role under all three conditions tested in the current study. Highest yielding lines under drought at jointing had high grain numbers, and lines that did well under full irrigation due to high grain numbers were also the best performers under drought at jointing (high‐yielding well‐watered and jointing drought; Figure 3d,e, Figure 4). Hence, selecting for grain number—which is relatively straightforward to estimate from yield and average kernel weight—is a good strategy for combining good performance under drought at jointing and yield potential. This confirms recent work in durum wheat (Vahamidis, Karamanos, & Economou, 2019) that presented the hypothesis that future yield improvements, especially for arid and semiarid environments, should be reached by increasing the capacity for setting grains. Combining high‐yield potential with resilience under drought at anthesis, however, also requires a stronger element of determinants of grain size (high‐yielding well‐watered and anthesis drought; Figure 3a,c, Figure 4). Interestingly, high‐yielding individuals in all three conditions had higher grain numbers than high‐yielding individuals across one or more conditions (Figure 4b). Overall, grain number and grain number retention is the most significant trait to select for drought tolerance. Large variation for both genetic determinants of grain number and size can be found in the population, hence providing the opportunity to combine both for yield stability, notwithstanding the inevitable trade‐off between these traits under resource‐limited conditions.

In this study, high‐yielding individuals within this population show that high leaf sugars are indicative of higher grain numbers during drought at jointing, and higher overall yield during drought at anthesis (Tables 1, 3), while the opposite is true for lower‐yielding lines where low leaf sugars during drought at jointing and anthesis indicate lower overall yields. Accumulation of sugars in drought‐stressed tissues is a survival mechanism common in extremely drought‐tolerant plants (Bernacchia & Furini, 2004). This may indicate that the accumulation of sugars in these high‐yielding wheat varieties aids survival during a drought event, and may provide energy for growth once the drought sink is relived (Fernández‐Marín et al., 2020). Carbohydrate metabolism such as starch biosynthesis and the breakdown of sucrose through sucrose synthase have been shown to be improved in the grain of wheat varieties (Paul, Watson, & Griffiths, 2019), but other processes such as signaling that determine grain numbers may still have room for optimization. Leaf sugars could act as a marker for high grain number with drought; that is, higher sugars would be a marker for higher yields. When looking at specific sugars in high‐yielding lines, high leaf sucrose after recovery from drought at jointing is associated with higher yields and grain numbers, while for drought at anthesis, higher grain fructose at drought and lower grain sucrose at recovery are associated with higher TGW (Figure 5). In low‐yielding lines, lower leaf sucrose at recovery after drought at jointing and lower grain fructose during drought when drought is applied at anthesis are both associated with lower grain numbers (Figure 6). This could suggest that recovery of photosynthesis and production of sugar after drought is a factor in enabling high grain numbers. Grain size (TGW) is also influenced by carbon availability through an influence on cell number and cell size and the subsequent filling of these cells by an active canopy (Brinton & Uauy, 2019).

In associating other traits under drought with yield, there was no relationship between leaf RWC and yield under either drought treatment (Figure S2) nor with recovery of RWC after drought (data not shown). Hence, neither avoidance nor tolerance mechanisms (Thiry, Chavez Dulanto, Reynolds, & Davies, 2016) appear to be favored regarding yield. It is unlikely therefore that selecting for RWC under drought would be a useful selection strategy. In some genotypes, RWC was lower at recovery than values recorded in drought (Figure 1), which indicated that those genotypes may hold onto water during drought as a drought‐avoidance mechanism (Yue et al., 2006). Higher leaf RWC could reflect root traits, where previous work has indicated that the rate of root growth and angle are key factors contributing to water uptake (Christopher et al., 2013; Rogers & Benfey, 2015) although roots traits were not determined in this study.

Increases in proline content have long been associated with drought stress (Hong‐Bo et al., 2006; Khamssi, 2014) with its role primarily linked to osmotic adjustment (Živčák, Repková, Olšovská, & Brestič, 2009) and membrane stabilization (Hayat et al., 2012). Our study shows that an increase in proline content during drought is negatively correlated with yield components in both trials. Mwadzingeni, Shimelis, Tesfay, and Tsilo (2016) have recently demonstrated that proline contents in flag leaves of drought‐stressed wheat are negatively correlated with TGW but not with yield. In contrast, our study shows that in a drought‐tolerant wheat population, higher leaf proline content during drought and recovery is associated with higher yield losses. Grain proline content during drought at anthesis was associated with higher TGW loss (Tables 3, 4, Figure S2). Recently, Alexander, Wendelboe‐Nelson, and Morris (2019) showed that transgenic drought‐tolerant barley has increased proline content in the leaves but provided no data on final yield of these plants. Our data suggest that proline may be acting as an osmolyte to preserve tissues, but this does not improve yield. Nor does proline aid in the optimal resumption of nonstressed plant metabolism after drought is alleviated. Proline accumulation may be an indication that tissue is drought‐stressed and may be related to overall survival under long‐term drought. However, proline may divert carbon and nitrogen away from productive processes, and in terms of productivity under drought, proline does not appear to act in a positive manner. Instead, proline content could be a suitable negative marker for high‐yielding drought‐tolerant wheat.

Overall, the study has provided a wealth of genetic diversity for drought resilience and yield potential, grain numbers, and TGW with the opportunity now for provision of lines for further crosses and for dissection of the genetic and mechanistic bases for combining yield potential with yield resilience to drought pre‐ and postanthesis. The most useful lines for further crosses are likely to be between those demonstrating high yields across all three conditions and those with high yields in two of the three treatments to see whether high grain number can be combined with filling grains to a larger size. Of course, there may be a limit to the trade‐off between grain size and number. Furthermore, there is potential of leaf and grain sugar measurement to be used as markers for high‐yielding wheat varieties. For genes and mechanisms, it will be interesting to compare extreme performers for grain number and size and whether genes already known to be linked to these traits such as in the trehalose pathway (Lyra et al., 2020) vary in this population and could provide markers for selection.

## CONFLICT OF INTEREST

None declared.

## AUTHOR'S CONTRIBUTION

CAG, MPR and MJP conceived and wrote the project. MPR designed and managed the field drought trials and provided data from well‐watered treatments. CAG and MJP sampled the field experiments and CAG extracted and measured leaf and ear samples. CAG analysed data. CAG, MPR and MJP wrote the paper which all three authors approved.

## Supporting information

Figure S1

Figure S2

Table S1

Table S2‐S4

Supplementary Material
