# Supplementary figures and images for: Combining yield potential and drought resilience in a spring wheat diversity panel
Source: Food Energy Secur. 2020 Sep 18;9(4):e241. doi: 10.1002/fes3.241 (PMC7771037; doi:10.1002/fes3.241)

## SUPPLEMENTARY FIGURE 1

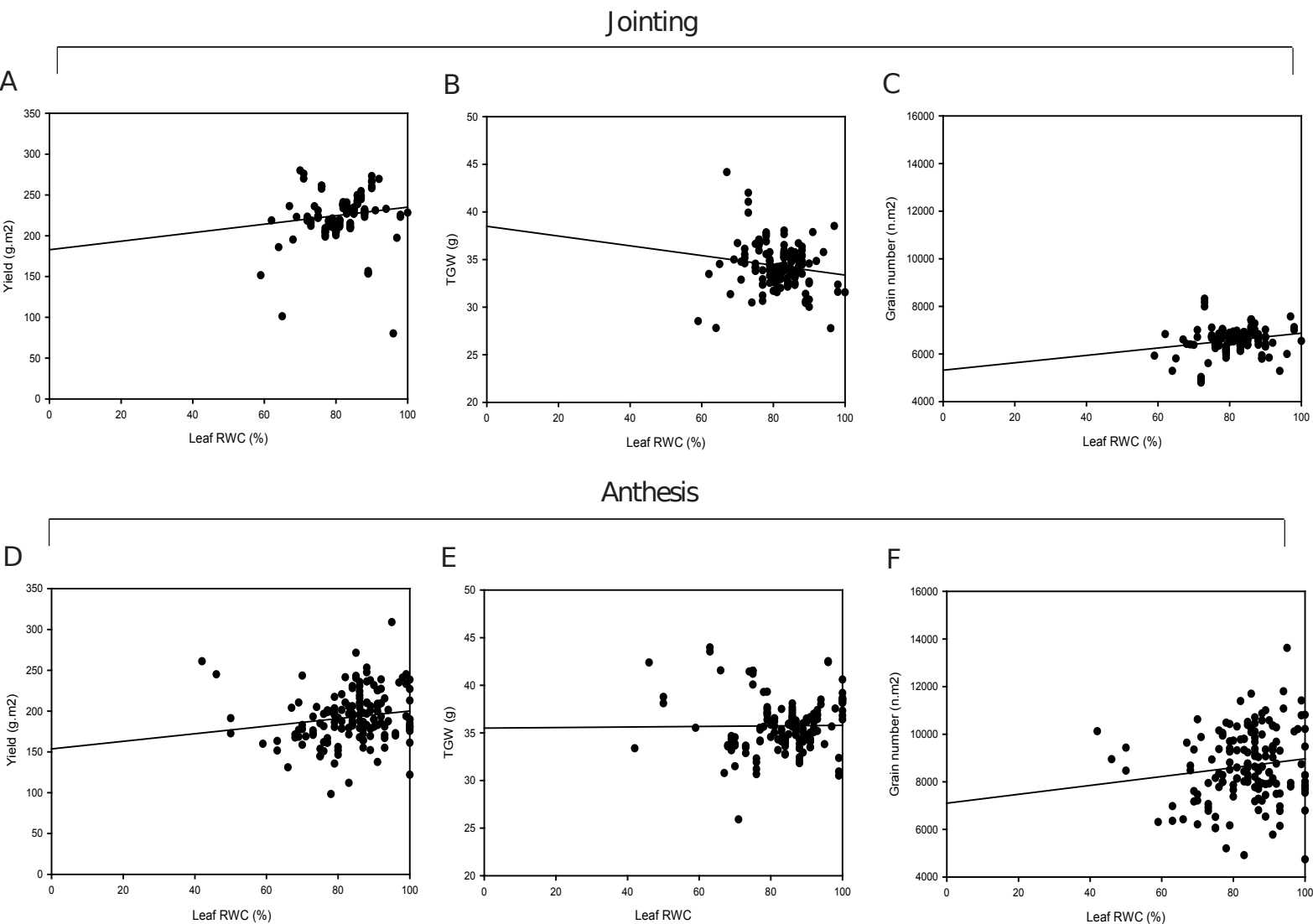

Supplement: Supplementary file 1 — Figure S1 [file FES3-9-e241-s001.pdf]

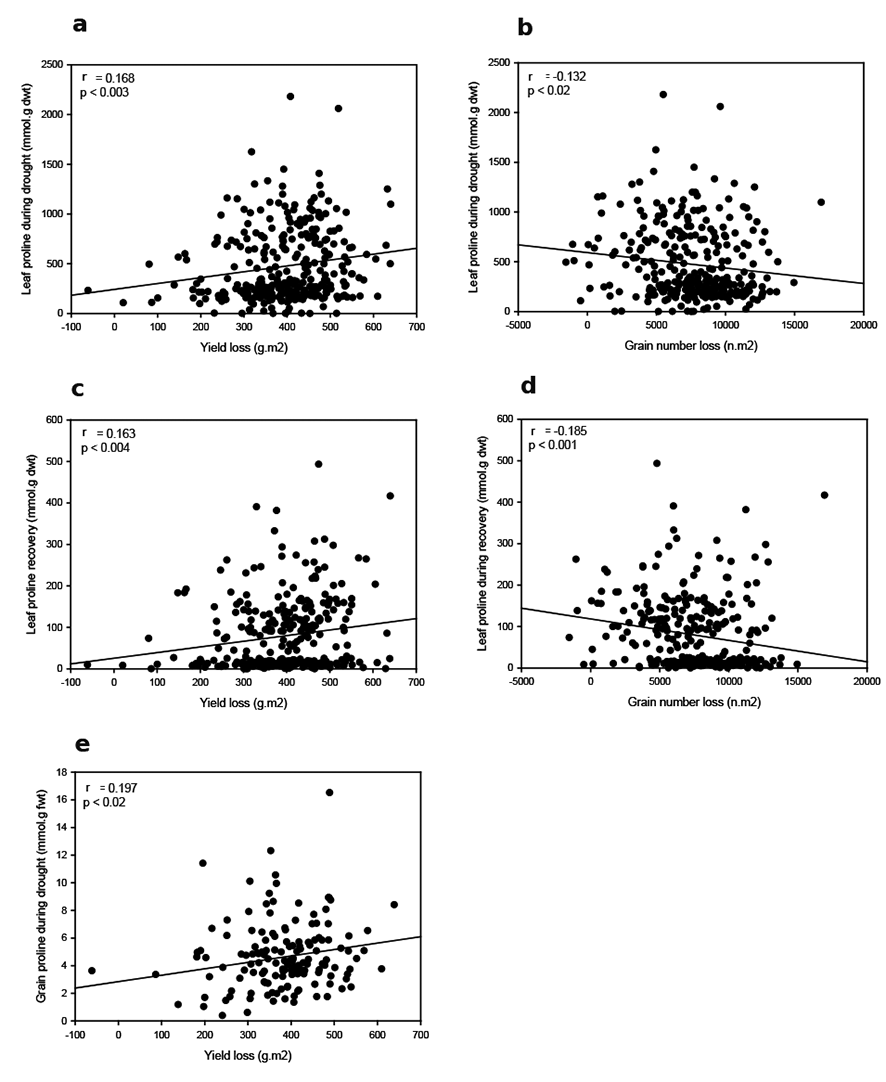

Supplement: Supplementary file 2 — Figure S2 [file FES3-9-e241-s002.tif]

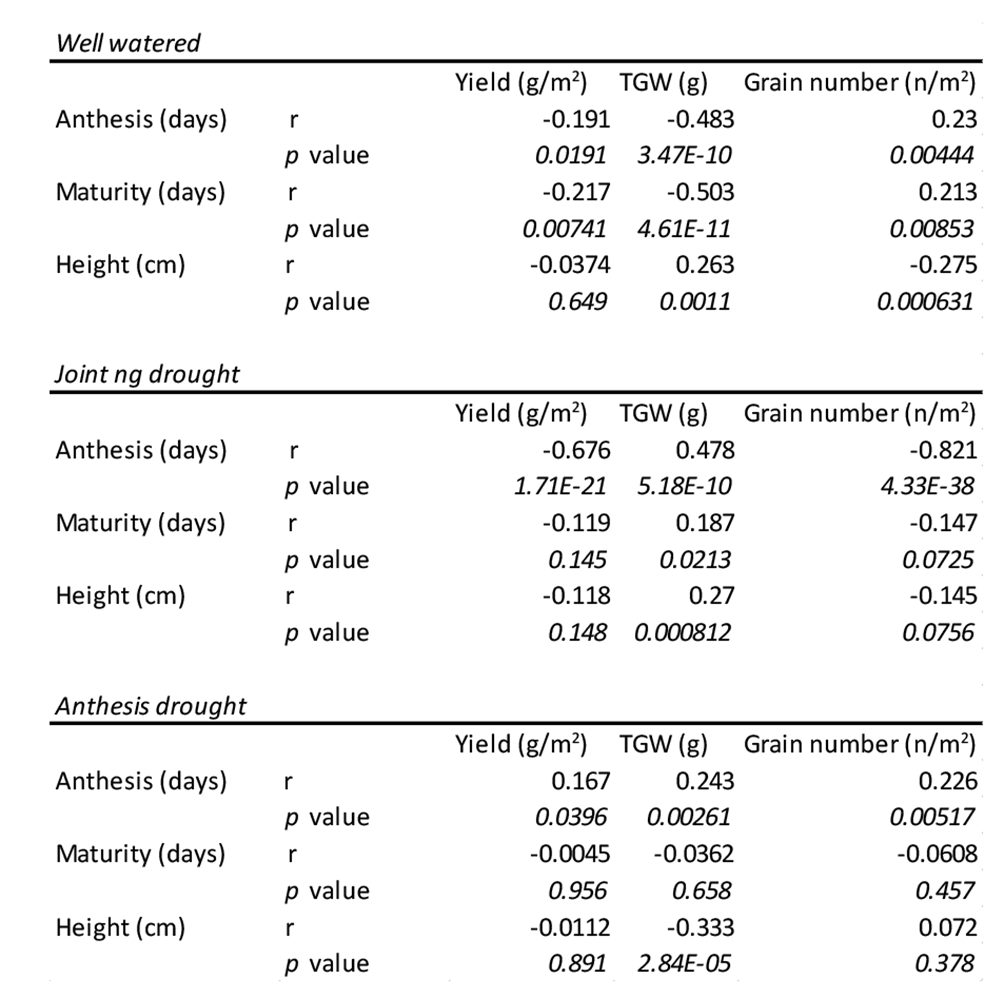

Supplement: Supplementary file 3 — Table S1 [file FES3-9-e241-s003.tif]
