# Supplementary material for: Combining yield potential and drought resilience in a spring wheat diversity panel
Source: Food Energy Secur. 2020 Sep 18;9(4):e241. doi: 10.1002/fes3.241 (PMC7771037; doi:10.1002/fes3.241)
Supplement: Supplementary file 5 — Supplementary Material [file FES3-9-e241-s005.docx]

**Supplementary data**

**Table S1:** Pearson product moment correlations between yield components and phenology data in well watered, jointing drought and anthesis drought field trials. n = 150.

**Table S2**: Well watered field trial yield component values of each diversity panel individual ordered by highest yield.

**Table S3**: Jointing drought field trial yield component values of each diversity panel individual ordered by highest yield.

**Table S4**: Anthesis drought field trial yield component values of each diversity panel individual ordered by highest yield.

**Figure S1**: Relationship between leaf relative water content (RWC) at drought and yield, TGW and grain numbers during both drought at jointing and drought at anthesis trials.

Pearson product moment correlations between leaf RWC and yield (a), TGW (b) and grain number (c) after drought at jointing, and leaf RWC and yield (d), TGW (e) and grain number (f) after drought at anthesis.

**Figure S2**: Relationship between proline content and yield component loss in both drought field trials.

Pearson product moment correlations between yield loss and leaf proline content during drought (a), recovery (c), grain number loss and leaf proline content during drought (b), recovery (d), and between yield loss and grain proline during drought at anthesis (e). Line denotes trendline, n = 300 (a,b,c,d), n = 150 (e).
